# BTK-Inhibitor Loaded Polymeric Nanoparticles Alleviate Systemic Lupus Erythematosus by Targeting Elimination of Autoreactive BAFFR^high^ B Cells

**DOI:** 10.3390/ijms27020729

**Published:** 2026-01-11

**Authors:** Yamin Zhang, Jingjing Wen, Biling Jiang, Hao Jiang, Jian Xu, Juan Tao

**Affiliations:** 1 Department of Dermatology, Union Hospital, Tongji Medical College, Huazhong University of Science and Technology, Wuhan 430022, China; 2018xh0170@hust.edu.cn (Y.Z.); wenjingjing816@163.com (J.W.); jiangbilingyo@163.com (B.J.); 2 Hubei Engineering Research Center for Skin Repair and Theranostics, Wuhan 430022, China; 3 Hubei Engineering Research Center for Biomaterials and Medical Protective Materials, School of Chemistry and Chemical Engineering, Huazhong University of Science and Technology, Wuhan 430074, China; hustjh@hust.edu.cn; 4 Institute of Hematology, Union Hospital, Tongji Medical College, Huazhong University of Science and Technology, Wuhan 430022, China

**Keywords:** Evobrutinib, anti-BAFFR antibody, systemic lupus erythematosus, nanoparticle, autoreactive B cell

## Abstract

Systemic lupus erythematosus (SLE) is a chronic and refractory autoimmune disease characterized by multi-organ damage, for which reliably safe and effective treatment remains an unmet need. Autoantibodies, secreted by autoreactive B cells, deposition is the central pathogenesis of organ damage in SLE. Current studies reported B cell receptor and B cell activating factor (BAFF)-mediated signals regulate the activation and survival of B cells and production of autoantibodies. We showed that marginal zone B cells and CD11c^+^T-bet^+^ autoreactive B cells expressed higher levels of BAFF receptor and BTK in MRL/lpr mice. Here, a liposome-delivery system capable of targeting BAFFR^high^ autoreactive B cells by conjugating anti-BAFFR antibody on the surface of the PEG-liposomes and loading BTK-inhibitor ibrutinib (BTEL) was rationally designed. Notably, the BTEL nanoparticles could inhibit the survival and activation of B cells, and systemic administration of BTEL could alleviate the development of the lupus mouse model by decreasing the production of anti-dsDNA autoantibodies, along with reduced secretion of inflammatory cytokines and kidney damage, and without apparent side effects. These findings suggest the potential of BTEL in targeting autoreactive B cells, blocking signaling pathways, and improving the efficacy of BTK inhibitors, providing a promising therapeutic approach for SLE, while also reducing toxicity.

## 1. Introduction

Systemic lupus erythematosus (SLE) is a chronic and refractory autoimmune disease characterized by a high level of autoantibodies in serum and damage to multiple organs and tissues [1]. Recent studies proposed the three-stage hypothesis of SLE, in which autoreactive B cells escaped from tolerance, secreting autoantibodies and forming immune complexes (ICs), and triggering organ damage caused by ICs deposition, ultimately, indicating the central pathogenesis of autoreactive B cells in SLE [2,3]. Therefore, targeting the elimination of autoreactive B cells and producing autoantibodies may be a promising strategy to reverse SLE tissue and organ damage.

B-cell depletion therapies have been widely studied in the clinics, including anti-B-cell activating factor (BAFF) therapies (Belimumab), B-cell depletion therapies of anti-CD20 mAb (rituximab), and anti-Bruton tyrosine kinase (BTK, Evobrutinib), etc. [4]. While more than 40% of patients received belimumab therapy in the phase III clinical trials failed to complete an effective clinical response [5]. Rituximab, a mouse-human chimeric monoclonal antibody that targets CD20, showed promising efficacy in treating lupus nephritis in a phase II clinical trial, but the phase III clinical trials were terminated prematurely due to serious infections [6]. Therefore, developing more specific therapies against the autoreactive but not pan B cell subset and autoantibodies with improved efficacy and safety still remains a big challenge.

BAFF interacts with three specific receptors, BAFF receptor (BAFFR), B cell maturation antigen (BCMA), and transmembrane activator and cyclophilin ligand interactor (TACI), thereby constituting a complex system of the BAFF system [5,7]. BAFFR only binds to BAFF and its primary role is to mediate the survival and maturation of immature B cells [7]. Several studies demonstrated that compared with the follicular compartment, autoreactive B cells are enriched within the marginal zone (MZ) compartment and expressed higher levels of BAFFR, which might be an important marker of autoreactive B cells [8,9]. However, blocking the BAFF–BAFFR pathway alone may not be as forcefully effective as pan-B cell depletion therapies, given the fact that Belimumab depletes B cells through BAFF neutralization, and only achieved modest efficacy in some SLE patients in randomized clinical trials [10]. Previous studies reported that B cell receptor (BCR)- and BAFF-mediated signals coordinately regulate positive and negative selection of autoreactive B cells, and are essential for activation, survival, and maturation of B cells and antibody secretion [8,11]. BTK is the key signal molecule and kinase involved in the intracellular signaling of BCR. It is essential for B-cell maturation, survival, and autoantibody production. Surprisingly, Evobrutinib, a highly selective, orally administered BTK inhibitor, failed to show a treatment effect versus placebo in double-blind, randomized, phase 2 trials for SLE and multiple sclerosis [12,13]. The underlying reason might be related to its short half-life (about 1 h), low bioavailability (<5% in rats and <25% in mice), restricted by hepatic and possibly intestinal first-pass metabolism, which severely hinders its efficient drug delivery into tissues and B cells, where BTK works [14]. As a result, higher dosages may be used to improve the efficacy, along with unwanted dose-related side effects, such as abnormal liver function. Therefore, a suitable carrier is a prerequisite for targeting the delivery of the BTK inhibitor into BAFFR^high^ autoreactive B cells.

Fortunately, the development of nano-based delivery technology made it possible to achieve the above-mentioned hurdles. Among various NP-based drug delivery systems, liposomes are initially known as carriers for diversified diagnostic and therapeutic agents, which have exhibited excellent safety and biocompatibility [15]. Herein, we developed a liposome-delivery system for targeting BAFFR^high^ autoreactive B cells by conjugating anti-BAFFR antibody on the surface of the PEG-liposomes for BTK-inhibitor Evobrutinib delivery. We showed that MZ B cells and CD11c^+^T-bet^+^ autoreactive B cells expressed higher levels of BAFFR and BTK in MRL/lpr mice, and the synthesized anti-BAFFR antibody conjugated to Evobrutinib Liposome (BTEL) could be phagocytized by MZ B cells effectively by binding to the BAFFR expressed on cells. Then, the therapeutic effect of BTEL NPs in lupus mice was investigated in vitro and in vivo. Notably, the BTEL could inhibit the survival and activation of B cells in vitro, and systemic administration of BTEL NPs could alleviate the development of model mice of lupus by decreased production of anti-dsDNA autoantibodies along with reduced systemic inflammation and kidney damage. Overall, by targeting the delivery of BTK inhibitors into autoreactive B cells, the BTEL NPs realized BAFFR- and BCR-signal pathway blocking simultaneously, inhibiting the survival and activation of autoreactive B cells and improving the bioavailability of BTK inhibitors, which may provide a promising strategy for the treatment of SLE.

## 2. Results and Discussion

### 2.1. The Expression of BAFFR and BTK Was Higher in MZ B Cells and CD11c^+^T-bet^+^ Autoreactive B Cells from MRL/lpr Mice

**Figure 1:**
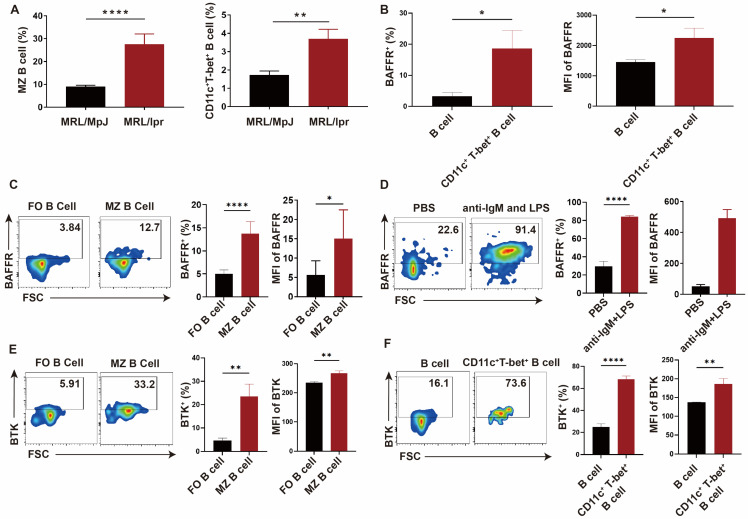
**BAFFR and BTK express higher in Marginal Zone B cells and CD11c^+^T-bet^+^ B cells in the spleen of MRL/lpr mice.** (**A**) Statistic graph of the percentage of Marginal Zone (MZ) B cells and CD11c^+^T-bet^+^ B cells in MRL/lpr mice compared with MRL/MpJ. (**B**) The percentage and median fluorescence intensity of BAFFR in CD11c^+^T-bet^+^ B cells in MRL/lpr mice. (**C**) The representative graph, statistic percentage, and median fluorescence intensity of Marginal Zone B cells compared with Follicular B cells in the spleen of MRL/lpr mice by flow cytometry. (**D**) The representative graph, statistic percentage and median fluorescence intensity of BAFFR in primary B cells purified of the spleen of MRL/lpr mice in vitro after undergoing anti-IgM and LPS stimulation for 24 h by flow cytometry. (**E**,**F**) The representative graph, statistic percentage, and median fluorescence intensity of BTK in MZ B cells (**E**) and CD11c^+^T-bet^+^ B cells (**F**) in the spleen of MRL/lpr mice by flow cytometry. Error bars represent the mean ± SD. * *p* < 0.05, ** *p* < 0.01, **** *p* < 0.0001.

MZ B cells have been reported to have increased populations of autoreactive B cells in several murine models, and positively correlate with pathological anti-dsDNA IgG production [16]. Flow cytometric analysis of B-cell subpopulation showed that the proportion of CD21^high^ CD23^low^ MZ B cells was increased in MRL/lpr mice (Figure S1 and Figure 1A). Especially, CD11c^+^T-bet^+^ B cells, a subset of autoimmune B cells predominantly found in the MZ region [17,18], was also expanded in MRL/lpr mice compared to wild-type controls (Figure 1A). Therefore, a therapeutic strategy targeted at these autoreactive cells could be a promising method.

BAFFR is the main binding receptor for BAFF, and its binding promotes B-cell survival, proliferation, antigen presentation, and B-cell differentiation into plasma cell [19]. Thus, we investigated whether BAFFR can be a suitable marker for targeting in these autoimmune cells. Importantly, the autoreactive subset CD11c^+^T-bet^+^ B cells and MZ B cells of MRL/lpr mice expressed higher levels of BAFFR compared to total B cells and follicular (FO) B cells, respectively (Figure 1B,C). Additionally, when primary mouse B cells purified from the spleen of MRL/lpr mice were cultured with B cell stimulation (Anti-IgM and LPS) [20,21], the percentage of BAFFR positive cells increased from 29.35 ± 5.58 to 83.93 ± 1.42, as demonstrated in Figure 1D. These results indicate that BAFFR could be a more specific target against the autoreactive B cell subset. Furthermore, the survival of several B cell subsets critically depends on the synergy of BAFFR and BCR signals, especially BTK [22]. In the lupus mouse model, we found that the percentage and median fluorescence index of BTK-positive cells were also significantly increased in MZ B cells and the CD11c^+^T-bet^+^ B cell subset (Figure 1E,F). Considering the overlap and higher expression of BTK and BAFFR in autoimmune B cells, NPs with anti-BAFFR modification and highly specific BTK inhibitor Evobrutinib loading were constructed. These NPs could be an effective way to target pathogenic B cells while reducing the toxicity and side effects of BTK in the lupus mouse model.

### 2.2. Preparation and Characterization of BTEL

**Figure 2:**
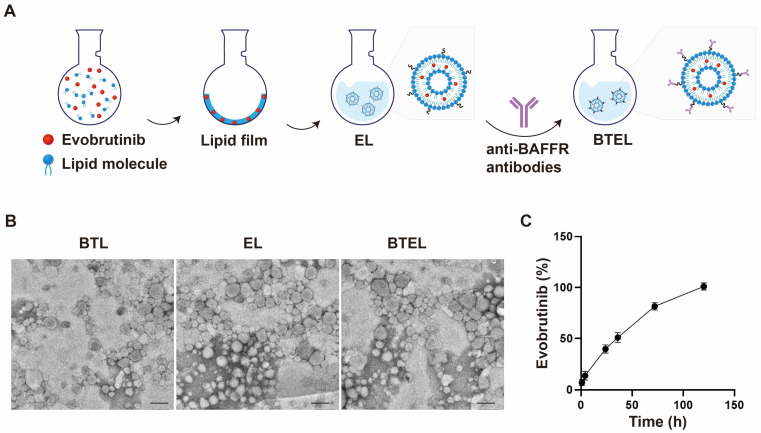
**Characterization of the NPs.** (**A**) Schematic illustration of BTEL. (**B**) SEM images of nanoparticles. (**C**) Drug release curves of BTEL. BTL, Anti-BAFFR antibody conjugated Liposome; EL, Evobrutinib Liposome; BTEL, Anti-BAFFR antibody conjugated Evobrutinib Liposome. Scale bar, 200 nm.

In order to target and further deplete autoimmune B cells, anti-BAFFR antibody-decorated BTEL consisting of cholesterol, lecithin, DSPE-PEG2000-COOH, and Evobrutinib was constructed through a thin film hydration method (Figure 2A). The presence of DSPE-PEG2000 endowed BTEL with excellent stabilization and prolonged circulation time in the blood [23]. The size distribution and zeta potential of blank Anti-BAFFR antibody conjugated Liposome (BTL), Evobrutinib Liposome (EL), and BTEL are summarized in Table 1. The average sizes of BTL, EL, and BTEL were 124.4, 123.2, and 125.7 nm, respectively (Table 1). Interestingly, the polydispersity index (PDI) of all the liposomes was below 0.2, indicating a relatively homogenous system. Compared to EL, the zeta potential of BTL and BTEL decreased, confirming the successful decoration of Anti-BAFFR antibody. Meanwhile, the morphologies of BTL, EL, and BTEL were investigated by SEM measurement (Figure 2B). The SEM images revealed that BTL, EL, and BTEL displayed a spherical structure. In addition, the loading efficiency and encapsulation efficiency of Evobrutinib in BTEL were 8.68 ± 0.03% and 32.63 ± 0.20%, respectively. Notably, the anti-BAFFR modification rarely changed the amount of Evobrutinib in the liposomes. Furthermore, the in vitro drug release behavior of BTEL in PBS (0.01 M, pH 7.4) was determined by a standard Evobrutinib calibration curve (Figure 2). As illustrated in Figure 2C, approximately 80% of Evobrutinib is released gradually from the BTEL over a 72 h period, indicating the drug’s sustained stability in the systemic circulation. These nanoparticles are engineered to selectively target autoimmune B cells through BAFFR recognition and facilitate the intracellular delivery of Evobrutinib. This targeted delivery approach minimizes drug leakage within a defined temporal window.

### 2.3. BTEL Inhibit the Viability and Activation of B Cells Through BAFFR In Vitro

**Figure 3:**
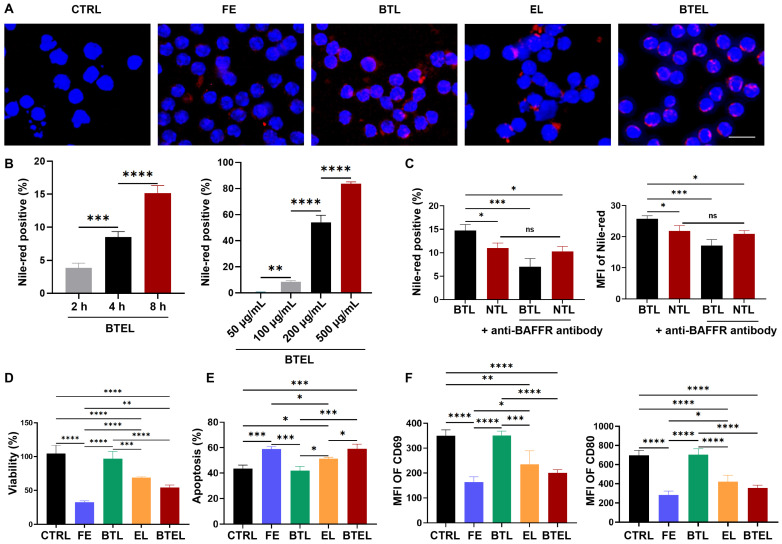
**Cell uptake, targeting performance, and anti-inflammatory impact on B cells post BTEL treatment in vitro.** (**A**) The representative immunofluorescence micrographs of cellular uptake in B cells after different treatment of 4 h. (**B**) Cellular uptake at different time of 100 μg/mL BTEL and different concentrations in 4 h. (**C**) Cellular uptake and MFI before and after BAFFR block in Marginal Zone B cell. (**D**) CCK-8 analysis of B cells viability measured at different treatment in 24 h. (**E**) Flow cytometry analysis of apoptosis measured after different treatment in 24 h. (**F**) Flow cytometry analysis of the percentages of CD69− and CD80− of B cells after different treatment in 24 h. CTRL, PBS Control; FE, Free Evobrutinib; BTL, Anti-BAFFR antibody conjugated Liposome; NTL, Non-Targeted Liposome; EL, Evobrutinib Liposome; BTEL, Anti-BAFFR antibody conjugated Evobrutinib Liposome. Scale bar, 10 μm. Error bars represent the mean ± SD. ns, not significant, * *p* < 0.05, ** *p* < 0.01, *** *p* < 0.001. **** *p* < 0.0001.

To ascertain the ability of BTEL to target BAFFR on autoimmune B cells, we initially investigated the cellular uptake of Nile-red-labeled BTEL across varying dosages and time durations. Cellular fluorescence analysis revealed enhanced cell uptake within the BTEL group (Figure 3A). Furthermore, flow cytometry analysis revealed a time- and concentration-dependent increase in BTEL uptake. At a fixed concentration of 100 μg/mL, intracellular fluorescence intensity rose from 3.8 ± 0.7% at 2 h to 15.2 ± 1.1% at 8 h. Similarly, under a 4 h incubation, increasing BTEL concentrations (50–500 μg/mL) led to a marked escalation in uptake from 0.5 ± 0.2% (50 μg/mL) to 83.7 ± 1.5% (500 μg/mL) (Figure 3B and Figure S2A).

Next, we assessed the cellular uptake of NPs in the presence or absence of anti-BAFFR modification in MZ B cells and FO B cells (Figure 3C and Figure S2B). The results showed a significant increase in uptake when NPs were modified with the anti-BAFFR antibody in MZ B cells, while no significant difference was observed in FO B cells. To further investigate whether BTL could be phagocytized by binding to BAFFR, a BAFFR blocking test was conducted in vitro. In BTL groups, the percentage of Nile red-positive cells decreased from 14.73 ± 1.28 to 7.01 ± 1.75 when treated with anti-BAFFR antibody. However, no significant difference was observed in the non-targeted liposome (NTL) groups (Figure 3C). No similar phenomenon was observed in FO B cells (Figure S2B). These results indicate that BTL can effectively target MZ B cells compared to NTL, and cellular uptake is mainly mediated by binding to BAFF receptors.

To further elucidate the effect of BTEL on B cells, cell death was evaluated. CCK8 analysis showed that BTEL induced cell death in a time-dependent manner (Figure 3D). Notably, treatment with free Evobrutinib (FE) resulted in a decrease in cell viability by 32.65 ± 1.88%, while BTEL and EL reduced viability by 54.03 ± 3.67% and 67.56 ± 1.27%, respectively. The lowest cell viability in the FE group was probably related to the small molecular weight, which can be phagocytized by the cell easily [24]. Additionally, cell apoptosis was identified using flow cytometry, yielding results akin to those of the CCK8 assay, indicating apoptosis as the primary mode of cell demise (Figure 3E). Moreover, we showed that treatment with BTEL resulted in much lower expression of CD69 and CD80, both surface markers of activated B cells, compared to PBS (Figure 3F). Based on the aforementioned results, these nanoparticles demonstrate the capacity to efficiently eliminate B cells and hinder their activation.

### 2.4. BTEL Effectively Inhibits the Progression of MRL/lpr Mice

**Figure 4:**
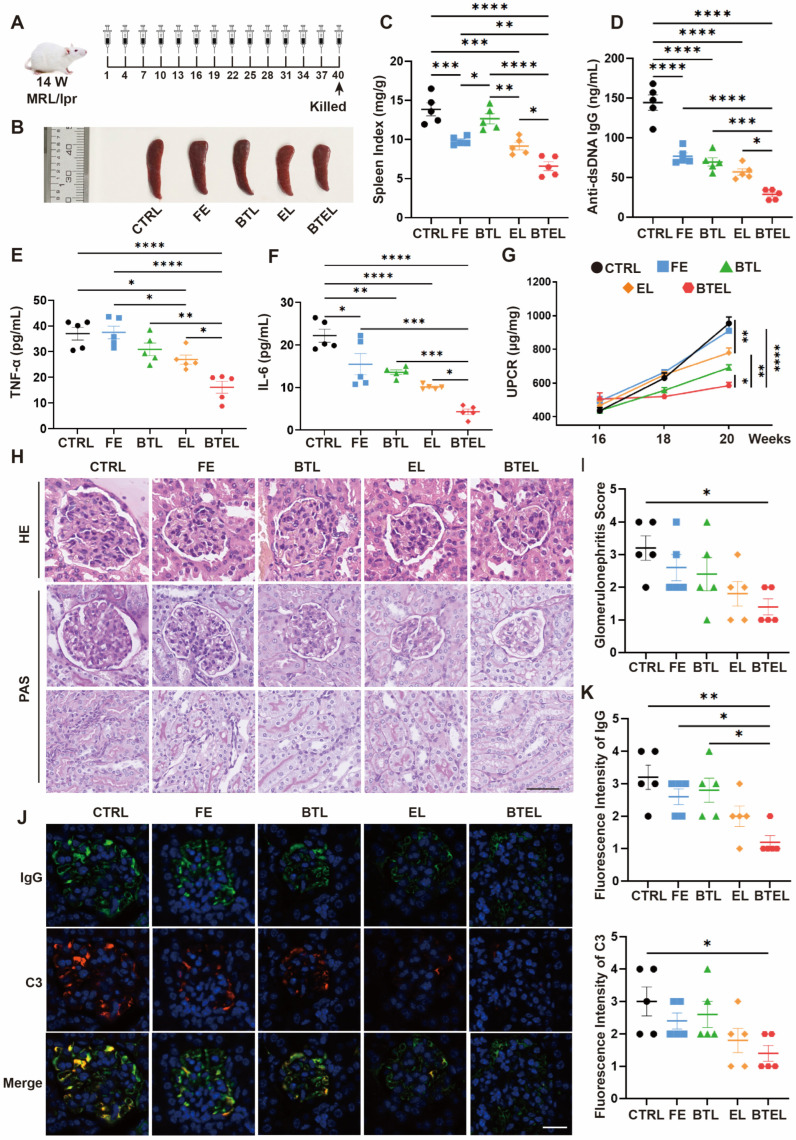
**BTEL effectively inhibits the progression of MRL/lpr mice.** (**A**) Schema for the animal experiment (*n* = 5/group). (**B**) The representative micrographs of mice spleen at the age of killed. Spleen index of lupus mice treated with CTRL, FE, BTL, EL and BTEL was measured by the end of the experiment (at the age of 20 weeks). (**C**) Spleen index was calculated as the ratio of spleen weight (mg) to body weight (g). (**D**) Serum concentrations of anti-dsDNA IgG at the age of killed. (**E**) Serum concentrations of TNF-α at the age of killed. (**F**) Serum concentrations of IL-6 at the age of killed. (**G**) The UPCR was obtained by measuring the urine protein and creatinine concentrations during the animal experiment. (**H**) The representative micrographs of H&E and PAS stain in kidney at the age of killed. (**I**) The glomerulonephritis score was assessed. (**J**) The representative immunofluorescence micrographs of the deposition of IgG and complement 3 in the glomeruli (green, IgG; red, C3; blue, nucleus). The fluorescence intensity of IgG and C3 (**K**) was assessed. CTRL, PBS Control; FE, Free Evobrutinib; BTL, Anti-BAFFR antibody conjugated Liposome; EL, Evobrutinib Liposome; BTEL, Anti-BAFFR antibody conjugated Evobrutinib Liposome. UPCR, urine protein-to-creatinine ratio. Scale bar, 20 μm. Error bars represent the mean ± SD. * *p* < 0.05, ** *p* < 0.01, *** *p* < 0.001, **** *p* < 0.0001.

We further evaluated the effect of BTEL in vivo. Fourteen-week-old MRL/lpr mice were randomized and intravenously injected once every three days with Ctrl, FE, BTL, EL, and BTEL for up to 40 days (Figure 4A). As shown in Figure 4B,C, representative micrographs of the spleen and spleen index demonstrate that the size of spleens in BTEL-treated mice was notably reduced compared to control groups (Figure 4B). Furthermore, although the spleen index of lupus mice treated with FE, BTL, and EL appeared to be smaller, there was no statistically significant difference (Figure 4C).

At the end of the experimental period, the serum level of anti-dsDNA IgG was also monitored [25]. BTEL clearly reduced the levels of anti-dsDNA autoantibodies more significantly than FE (*p* < 0.05), and EL (*p* < 0.05) (Figure 4D), and the impact of BTK inhibitors alone on anti-dsDNA IgG was modest, consistent with previous findings [20]. This suggests that a subset of B cells can evade BTK inhibition and differentiate into plasma cells, highlighting the significance of the BAFF–BAFFR pathway in the survival and maturation of immature B cells [26]. Consequently, the combined addition of anti-BAFFR antibody modification resulted in a synergistic effect, leading to improved inhibition [27]. Additionally, as shown in Figure 4E,F, the IL-6 levels were remarkably reduced in MRL/lpr mice treated with FE, BTL, EL and BTEL. In comparison, no significant difference in TNF-α was observed in lupus mice treated with FE, BTL and EL at the time of kill when compared with control, indicating that FE, BTL and EL may not affect TNF-α secretion. Particularly, BTEL inhibited the production of TNF-α and IL-6 more efficiently than other groups, which is well in accordance with the results of Anti-dsDNA IgG level and reduced splenic enlargement. Moreover, lupus-related kidney damage remains one of the major factors limiting survival improvement in patients with this disease [28]. As shown in Figure 4G, urine protein-to-creatinine ratio (UPCR) gradually increased in lupus mice treated with PBS control and FE from the age of 16 weeks and peaked at the age of 20 weeks, indicating that FE could not delay lupus nephritis. At the age of 20 weeks, UPCR decreased in the groups treated with BTL, EL, and BTEL, with the most pronounced decrease observed in the BTEL-treated group. The reduction seen with BTL alone highlights the contribution of BAFFR blockade in mitigating renal injury, likely through limiting autoreactive B-cell survival [29].

To further test the therapeutic efficacy of BTEL on lupus nephritis, we performed a pathological examination of the kidneys of mice. The histology of BTEL-treated MRL/lpr mice demonstrated reduced glomerular enlargement in the H&E and PAS staining (Figure 4H,I). Additionally, Immunofluorescence staining of IgG and C3 in kidney sections was performed at the end of the experiment. BTEL most significantly reduced the deposition of IgG and C3 in the kidney compared to all other treatment groups (Figure 4J,K). This observation aligns with previous studies demonstrating that BTK inhibitors can delay the progression of nephritis in lupus model mice. Moreover, BTEL appears to be even more effective, possibly due to the anti-BAFFR antibody’s ability to enhance the sensitivity of B cells to drugs [20,30,31,32]. Therefore, BTEL more effectively mitigated the progression of systemic damage and kidney injury in lupus mice compared to FE and EL.

### 2.5. Treatment of BTEL Decreased the Percentage and Number of Autoreactive B Cells

**Figure 5:**
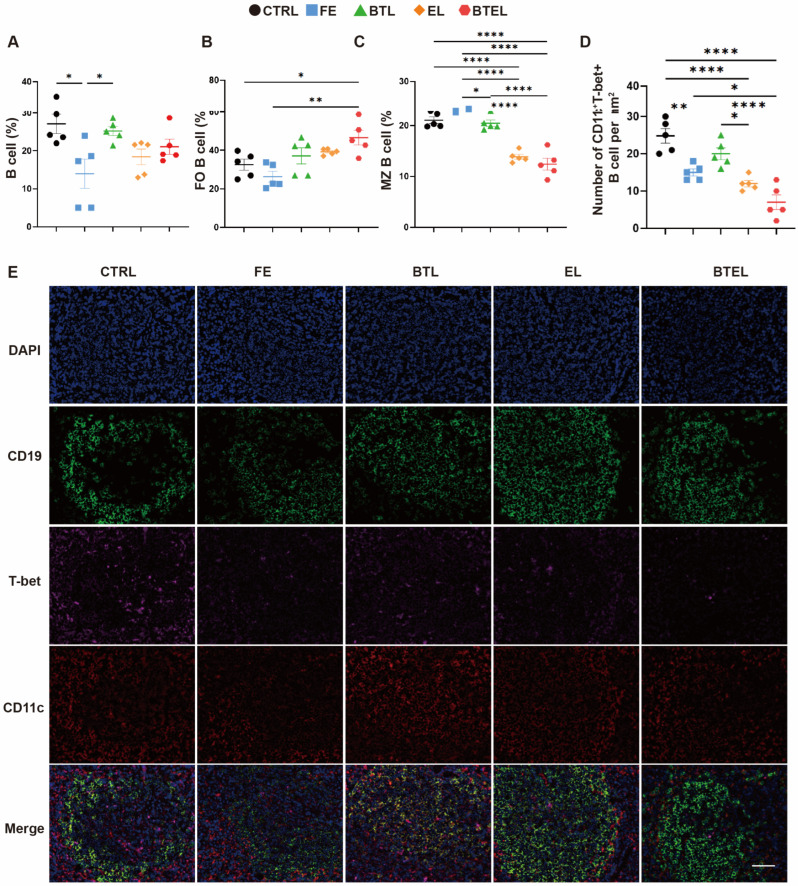
**Changes in B cells and subsets in the spleen of MRL/lpr mouse after NPs therapy.** Counts were taken of infiltrating cells per spleen, including the following: (**A**–**C**) The statistical percentage of total B cells (**A**), Follicular B cells (**B**), Marginal Zone B cells (**C**). (**D**,**E**) The statistical numbers (**D**) and representative immunofluorescence micrographs (**E**) of CD19^+^CD11c^+^T-bet^+^ cells in the spleen (green, CD19; pink, T-bet; red, CD11c; blue, nucleus; *n* = 5/group). CTRL, PBS Control; FE, Free Evobrutinib; BTL, Anti-BAFFR antibody conjugated Liposome; EL, Evobrutinib Liposome; BTEL, Anti-BAFFR antibody conjugated Evobrutinib Liposome. Scale bar, 50 μm. Error bars represent the mean ± SD. * *p* < 0.05, ** *p* < 0.01, **** *p* < 0.0001.

Considering the important role of B cells in SLE and in order to clarify the underlying mechanism of BTEL, we explored the effects of all treatments on the total number and the subtype of B cells in the spleens (Figure S3). We found that only treatment with FE resulted in a decrease in the total cell number compared to mice treated with PBS (Figure 5A). This suggests that while FE has a therapeutic effect on the lupus mouse model, it may have broad targets of action and can easily lead to side effects.

By conducting flow cytometric analysis of B-cell subpopulations, we noted that treatment with FE had no significant effect on the subset of MZ B cells, which is consistent with a previous study [20]. However, the treatments with EL and BTEL led to a significant decrease in the percentage of MZ B cells, from 20.58 ± 1.17 to 12.70 ± 1.13 and 14.88 ± 2.63, respectively. Importantly, BTEL not only decreased the proportion of MZ B cells but also increased the population of FO B cells (Figure 5B,C). This effect may be attributed to the liposomes prolonging drug cycle time, reducing off-target effects, effectively targeting autoreactive B cells, and creating synergistic effects with BAFFR and BTK [14,27,33]. In contrast, BTL alone did not induce significant changes in these B-cell subsets compared to the control, indicating that solely blocking the BAFF–BAFFR axis may be insufficient to reverse established compositional abnormalities in the spleen, despite its efficacy in reducing overall disease activity [34]. Furthermore, the alteration in CD19^+^CD11c^+^T-bet^+^ B cell population was analyzed through immunofluorescence staining, as demonstrated in Figure 5D,E, exhibiting a notable decrease in the BTEL group. These findings indicate that BTEL effectively reverses B-cell abnormalities and normalizes the proportions of splenic B-cell subtypes.

### 2.6. Systemic Impacts and Safety of BTEL

**Figure 6:**
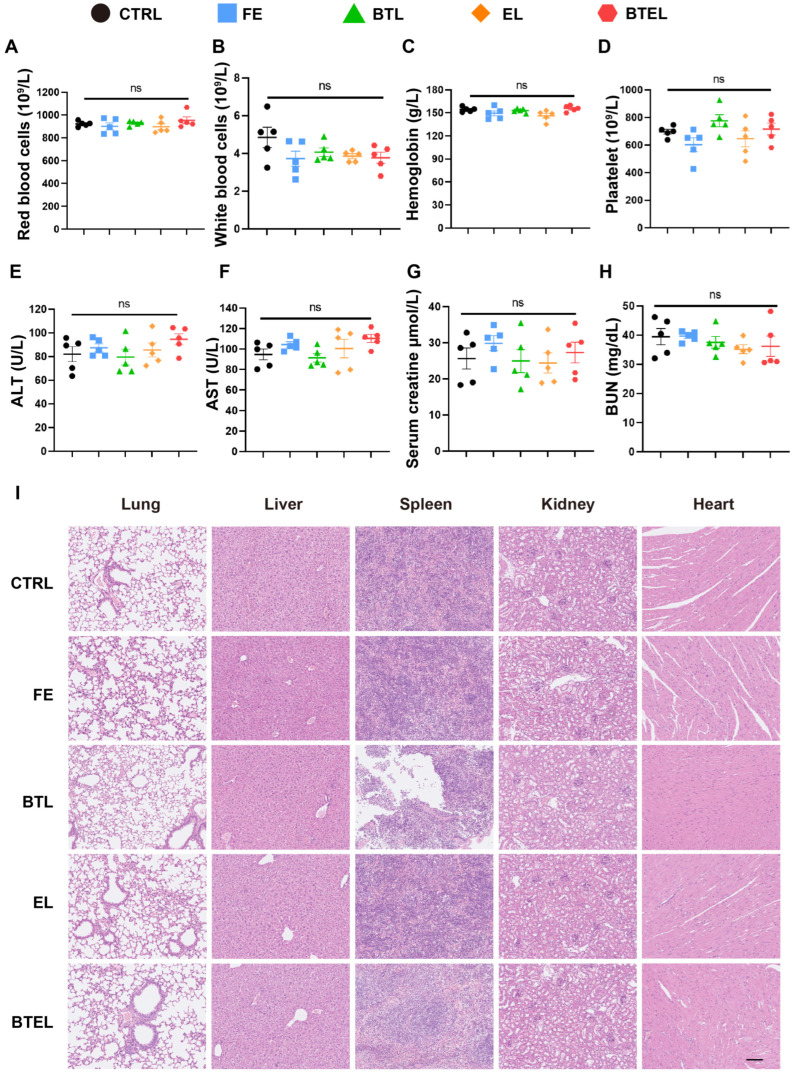
**Blood examinations and blood biochemistry analyses in the C57BL/6 mouse indicated no obvious pathological manifestations after the BTEL treatment regimen.** (**A**–**D**) Total red blood cell (**A**), total white blood cell (**B**), Hemoglobin level (**C**), and platelet count (**D**) were determined using a hematology analyzer. (**E**,**F**) Serum levels of ALT and AST were detected by a biochemical analyzer. (**G**,**H**) BUN and serum creatinine levels were also detected by a biochemical analyzer. (**I**) H&E staining of major organs (lung, liver, spleen, kidney, and heart), Scale bar, 100 μm. *n* = 5/group. Error bars represent the mean ± SEM. ns, not significant.

The systemic impacts and safety were assessed. At the end of the 7-day treatment period, the counts of white blood cells, neutrophils, monocytes, and lymphocytes were evaluated. No significant differences were observed between any of the treatment groups, indicating that the administration of BTEL did not affect the hematopoietic system (Figure 6A–D). Additionally, there were negligible changes in serum levels of ALT, AST, CREA, and BUN across all groups, suggesting that the dosage of BTEL used in this study did not induce acute liver or renal toxicities (Figure 6E–H). Furthermore, histological examination of major organs using H&E staining revealed no detectable tissue damage in any of the treatment groups (Figure 6I). This suggests that the administration of nanocarriers did not result in histopathological abnormalities. Taken together, these findings indicate the superior biocompatibility and safety of BTEL for in vivo treatment of lupus.

### 2.7. Limitations

This study has several limitations that should be considered. First, all experiments were conducted in a murine model of SLE. Although the MRL/lpr model is well-established, future validation using human SLE patient-derived cells or humanized models is necessary for clinical translation. Second, the detailed molecular mechanism underlying the synergy between BAFFR targeting and BTK inhibition, particularly regarding downstream signaling pathways, was not elucidated and warrants future investigation. Third, a direct comparison with a clinically approved anti-BAFF therapy (e.g., a belimumab analog) or a simple combination of free drugs was not performed. Such comparisons would further define the unique advantages of the nanoparticulate system. Finally, the sample size, while consistent with preliminary efficacy studies in this model, is relatively small. Confirmatory studies with larger animal cohorts are needed to strengthen the statistical robustness of the findings, particularly for highly variable parameters.

## 3. Materials and Methods

### 3.1. Materials

Anti-BAFF-R (mouse), mAb (9B9) was supplied by Adipogen Corp. (San Diego, CA, USA). Evobrutinib was purchased from Selleck Co. (Houston, TX, USA). Cholesterol, lecithin, and DSPE-PEG2000-COOH were from SunLipo Nano Tech (Shanghai, China).

### 3.2. Materials and Synthesis of BTEL

Evobrutinib-loaded Liposome, consisting of Evobrutinib, cholesterol, lecithin, and DSPE-PEG2000-COOH, was initially conducted by a thin film hydration method according to a previous report [23]. Then, the carboxyl groups on the surface of liposomes were activated by adding EDC and NHS, followed by co-incubation with anti-BAFFR antibodies. The free Evobrutinib and antibodies were removed through dialysis to obtain BTEL. Dynamic light scattering (DLS, ZEN3690; Malvern Panalytical, Melbourne, Malvern, UK) was used to determine the size and distribution of BTEL. Scanning transmission microscopy (SEM, Carl Zeiss G300 microscope, Dusseldorf, Germany) was used to display the morphology of BTEL.

### 3.3. B Cells Culture and Stimulation In Vitro

CD19^+^ B cells were purified from the spleens of MRL/lpr mouse using a mouse CD19+ B cell isolation kit (Miltenyi Biotec, Bergisch Gladbach, Germany), and a purity of higher than 98% purity was validated by flow cytometry. MZ B cells and Follicular (FO) B cells were purified by mouse MZ and FO B Cell Isolation Kit (Miltenyi Biotec). Purified cells were cultured at 10^6^ cells/mL for 1 to 3 days in culture medium containing RPMI 1640, 10% FCS, and 50 g/mL gentamycin (all from Gibco, Thermo Fisher Scientific, Waltham, MA, USA). For B cell stimulation, cells were cultured in vitro with 10 μg/mL Ag binding fragment [F(ab)^2^] -IgM (Anti-IgM) (Jackson ImmunoResearch Laboratories, West Grove, PA, USA), 1 μg/mL lipopolysaccharide (LPS; own production) for 24 h [21].

### 3.4. Nanoparticles Uptake Assay for B Cells In Vitro

B cells were seeded into 24-well plates at a density of 2.5 × 10^5^ cells/well. The cells were then incubated with Nile Red-BTEL (containing 50, 100, 200, or 500 μg/mL Evobrutinib) for 2, 4, or 8 h. Next, B cells were treated with PBS (CTRL), Free Evobrutinib (FE), Anti-BAFFR antibody conjugated Liposome (BTL), Evobrutinib Liposome (EL), Anti-BAFFR antibody conjugated Evobrutinib Liposome (BTEL) (200 μg Evobrutinib equiv./mL, respectively, for 8 h. Flow cytometry was then used to detect the Nile Red fluorescence. In the BAFFR block experiment, Anti-BAFFR antibody (AdipoGen, San Diego, CA, USA) was added into the culture of B cells at a concentration of 4 μg/mL to block BAFF. Purified MZ and FO B cells were pre-incubated with either or both of the blocking antibodies for 30 min and then co-incubated with Nile Red-BTL or Nile Red-NTL for 8 h. The positive percentage and fluorescence intensity of Nile red in B cells were measured with an LSR II flow cytometer (BD Biosciences, Franklin Lakes, NJ, USA).

### 3.5. Effects of BTEL on B Cells In Vitro

After being seeded into 96-well or 48-well plates, B cells were treated with either CTRL, FE, BTL, EL, BTEL, respectively (100 μg Evobrutinib equiv./mL) for 24 h. The viability status and apoptosis percentage of B cells were assessed by CCK-8 (Dojindo, Shanghai, China) and flow cytometry (BD LSR II, Franklin Lakes, NJ, USA) after incubation, respectively. The surface molecules, such as CD69 (Biolegend, San Diego, CA, USA) and CD80 (Biolegend, San Diego, CA, USA), on the B cells were analyzed by flow cytometry (BD LSR II, Franklin Lakes, NJ, USA).

### 3.6. Animals

Female MRL/lpr lupus-prone and MRL/MpJ control mice, aged 8 weeks and weighing 25–30 g, were purchased from Cavens Laboratory Animal Co., Ltd. (Changzhou, China). These mice were all raised in the Animal Center of the Huazhong University of Science and Technology, under specific pathogen-free conditions. All animal experiments met the requirements of the Guide for the Care and Use of Laboratory Animals of Huazhong University of Science and Technology and were approved by the Institutional Animal Care and Use Committee, Tongji Medical College, Huazhong University of Science and Technology.

### 3.7. Treatment Study In Vivo

After being raised to 14 weeks of age, MRL/lpr mice were randomly divided into five groups (*n* = 5/group) as follows: CTRL, FE, BTL, EL, BTEL. Drugs were administered by intraperitoneal injection in a 250 μL volume every 3 days for 40 days (20 μg Evobrutinib equiv./mice). The sample size (*n* = 5 per group) was determined based on previous interventional studies in the MRL/lpr model [25,35]. During this process, the weight of the mice was recorded. The titer of autoantibodies against dsDNA in the serum was determined by ELISA (Cusabio, Wuhan, China). The serum levels of TNF-α and IL-6 were measured using the cytometric bead array cytokine kit (BD, Franklin Lakes, NJ, USA). Urine was obtained by bladder massage. Urine protein and creatinine were detected by the Coomassie brilliant blue G-250 dyeing method and creatinine assay kits (Sigma-Aldrich, St. Louis, MO, USA), respectively. At the end of the treatment period, spleens were collected for flow cytometry analysis of B cells. CD19^+^CD11c^+^T-bet^+^ cells were detected by immunofluorescence staining (CD19, Abcam, Cambridge, UK; CD11c, CST, Danvers, MA, USA; T-bet, Proteintech, Wuhan, China). The other kidney and internal organs (including the heart, liver, and lungs) were fixed (using 4% paraformaldehyde) and embedded in paraffin.

### 3.8. Flow Cytometry Analysis

The mouse spleens were photographed and processed into a single-cell suspension. Dead cells were identified using a fixable viability dyeing (Biolegend, San Diego, CA, USA). An anti-CD16/32 blocking antibody (Biolegend, San Diego, CA, USA) was used to reduce non-specific binding before staining with other antibodies, including anti-mouse CD45, CD19, CD21, CD23, BAFF-R, and their isotype controls (all from Biolegend, San Diego, CA, USA). Stained cell suspensions were all analyzed by flow cytometry (BD LSR II, Franklin Lakes, NJ, USA).

### 3.9. Histological Analysis and Immunofluorescence of the Mouse Kidney

The hematoxylin-eosin (H&E) staining and periodic acid-Schiff (PAS) staining of the kidney were used to assess pathological damage to the kidney. Pathological change in the glomerulus was scored (0–4) by experienced nephrologists (0, normal; 1, 1–25% damage; 2, 26–50% damage; 3, 51–75% damage; and 4, >75% damage) as described in previous studies. The deposition of IgG and complement 3 (C3) was detected by immunofluorescence staining (IgG, Abcam, Cambridge, UK; C3, Santa Cruz Biotechnology, Santa Cruz, CA, USA). The fluorescence intensity of IgG and C3 was graded based on the stained area as a percentage (0, <1%; 1, 1–25%; 2, 26–50%; 3, 51–75%; and 4, >75%) [35].

### 3.10. Short-Term Toxicity

C57BL/6 normal female mice, aged 8 weeks, were randomly grouped (*n* = 5/group) and were administered with the indicated drugs as in an animal treatment experiment for 7 days. The body weights were recorded. At the end of the study, blood samples were collected. The serum levels of alanine aminotransferase (ALT), aspartate aminotransferase (AST), blood urea nitrogen (BUN), and serum creatinine were measured (Hitachi 7600, Tokyo, Japan). The visceral organs (heart, liver, spleen, lung, and kidney) were harvested and fixed for H&E staining.

### 3.11. Statistical Analysis

Statistical analysis was conducted using GraphPad Prism 9 software. Quantitative data were expressed as a mean value ± standard deviation (SD). An unpaired Student’s *t*-test was performed to analyze the statistical significance between two groups, and one-way ANOVA was used to do so among multiple groups. *p* < 0.05 was considered to indicate statistical significance. * *p* < 0.05, ** *p* < 0.01, *** *p* < 0.001 and **** *p* < 0.0001.

## 4. Conclusions

In this study, we successfully developed a targeted delivery system, which allowed specific delivery of BTK inhibitor Evobrutinib to BAFFR^high^ auto-reactive B cells, leading to a significant slowdown in the progression of lupus model mice. The anti-BAFFR antibody within BTEL contributed additively to its efficacy, as evidenced by the therapeutic activity of BTL alone, indicating that BAFFR engagement likely modulates B-cell survival and function beyond enhancing drug targeting. Together, the combined action of BAFFR targeting and BTK inhibition in BTEL produced a synergistic therapeutic outcome. We observed a reduction in the production of anti-dsDNA autoantibodies, decreased systemic inflammation, and attenuated renal injury, while without significant biological side effects. The mechanism underlying the therapeutic effects of BTEL in SLE involves a decrease in the percentage and number of auto-reactive B cells and an inhibition of inflammatory cytokines in the circulation. By specifically targeting and eliminating autoreactive B cells, this delivery system offers a promising strategy for the treatment of SLE and the mitigation of disease progression.

## Figures and Tables

**Table 1:** The physicochemical properties of the NPs.

	Average Size (nm)	PDI	Zeta Potential (mV)
BTL	124.4 ± 4.4	0.150 ± 0.002	−30.6 ± 9.99
EL	123.2 ± 3.8	0.148 ± 0.002	−14.6 ± 6.37
BTEL	125.7 ± 2.6	0.151 ± 0.012	−25.7 ± 5.15

## Data Availability

The original contributions presented in this study are included in the article/Supplementary Material. Further inquiries can be directed to the corresponding authors.

## Supplementary Materials

The following supporting information can be downloaded at: https://www.mdpi.com/article/10.3390/ijms27020729/s1.
