# Supplementary material for: BTK-Inhibitor Loaded Polymeric Nanoparticles Alleviate Systemic Lupus Erythematosus by Targeting Elimination of Autoreactive BAFFRhigh B Cells
Source: Int J Mol Sci. 2026 Jan 11;27(2):729. doi: 10.3390/ijms27020729 (PMC12841580; doi:10.3390/ijms27020729)
Supplement: Supplementary file 1 [file ijms-27-00729-s001.zip › ijms-4046070-supplementary.pdf]

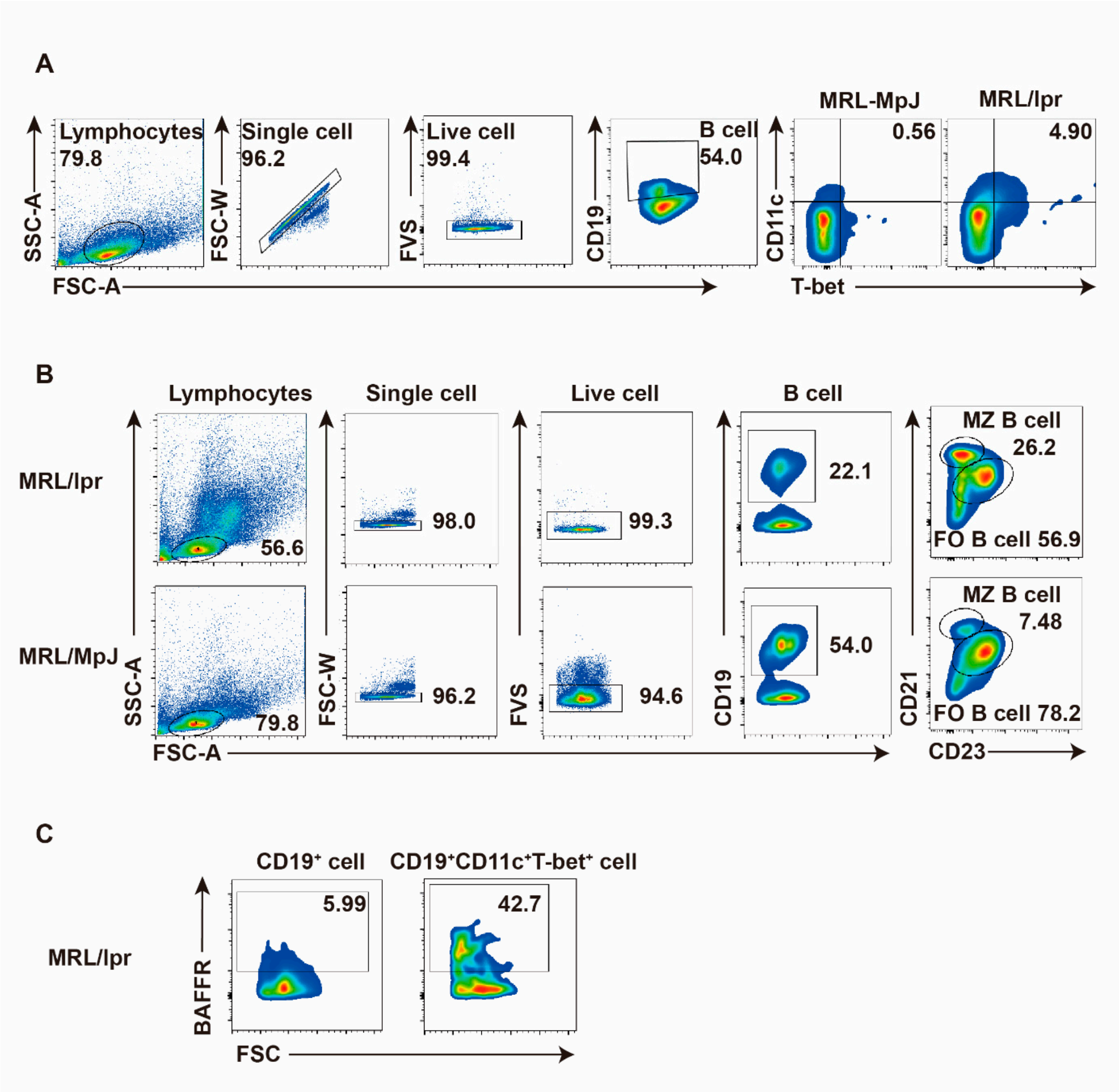

Figure S1. (A) The representative graphs of the percentage of CD11c^{+}T-bet^{+} B cells in MRL/MpJ and MRL/lpr mice; (B) Marginal Zone (MZ) B cells and Follicular (FO) B cells in MRL/MpJ and MRL/lpr mice by flow cytometry. (C) The representative graphs of the percentage of BAFFR in B cell (CD19^{+}) and CD11c^{+}T-bet^{+} B cells.

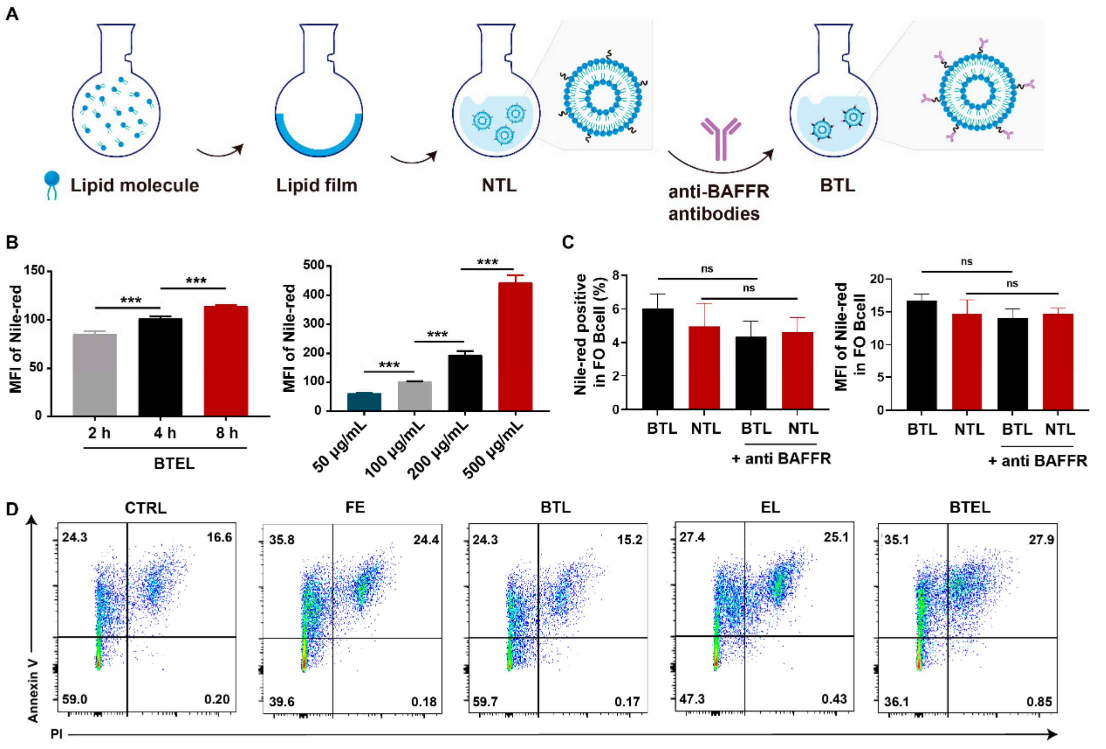

Figure S2. (A) Schematic illustration of BTL. (B) The mean fluorescence intensity (MFI) at different time of 100 μg/mL BTEL and different concentrations of BTEL in 4 h; (C) Cellular uptake and MFI before and after BAFFR block in Follicular B cells. (D) The representative graphs of apoptosis after different treatment in 24 h by flow cytometry. BTL, BAFFR-Targeted Liposome; NTL, Non-Targeted Liposome; Error bars represent the mean ± SD. ns, not significant, ***P < 0.001.

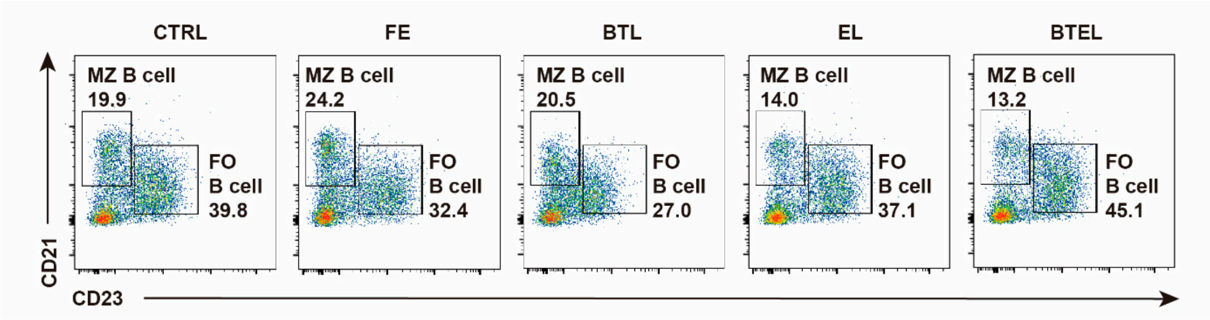

Figure S3. The representative graphs of flow cytometry analysis of Marginal Zone (MZ) B cells and Follicular (FO) B cells.
